# Association of Sarcopenia with a Poor Prognosis and Decreased Tumor-Infiltrating CD8-Positive T Cells in Pancreatic Ductal Adenocarcinoma: A Retrospective Analysis

**DOI:** 10.1245/s10434-023-13569-2

**Published:** 2023-05-16

**Authors:** Shigeto Masuda, Kohei Yamakawa, Atsuhiro Masuda, Hirochika Toyama, Keitaro Sofue, Yoshihide Nanno, Shohei Komatsu, Satoshi Omiya, Arata Sakai, Takashi Kobayashi, Takeshi Tanaka, Masahiro Tsujimae, Shigeto Ashina, Masanori Gonda, Shohei Abe, Hisahiro Uemura, Shinya Kohashi, Noriko Inomata, Kae Nagao, Yoshiyuki Harada, Mika Miki, Yosuke Irie, Noriko Juri, Maki Kanzawa, Tomoo Itoh, Takumi Fukumoto, Yuzo Kodama

**Affiliations:** 1 Division of Gastroenterology, Department of Internal Medicine, Kobe University Graduate School of Medicine, Kobe, Hyogo Japan; 2 Division of Hepato-Biliary-Pancreatic Surgery, Department of Surgery, Kobe University Graduate School of Medicine, Kobe, Hyogo Japan; 3 Department of Radiology, Kobe University Graduate School of Medicine, Kobe, Hyogo Japan; 4 Division of Diagnostic Pathology, Kobe University Graduate School of Medicine, Kobe, Japan

**Keywords:** Pancreatic ductal adenocarcinoma, Sarcopenia, Skeletal muscle index, Immune cells, Tumor microenvironment

## Abstract

**Background:**

Sarcopenia, defined as a loss of skeletal muscle mass and quality, is found in 30–65% of patients with pancreatic ductal adenocarcinoma (PDAC) at diagnosis, and is a poor prognostic factor. However, it is yet to be evaluated why sarcopenia is associated with poor prognosis. Therefore, this study elucidated the tumor characteristics of PDAC with sarcopenia, including driver gene alterations and tumor microenvironment.

**Patients and Methods:**

We retrospectively analyzed 162 patients with PDAC who underwent pancreatic surgery between 2008 and 2017. We defined sarcopenia by measuring the skeletal muscle mass at the L3 level using preoperative computed tomography images and evaluated driver gene alteration (*KRAS*, *TP53*, *CDKN2A*/p16, and *SMAD4*) and tumor immune (CD4^+^, CD8^+^, and FOXP3^+^) and fibrosis status (stromal collagen).

**Results:**

In localized-stage PDAC (stage ≤ IIa), overall survival (OS) and recurrence-free survival were significantly shorter in the sarcopenia group than in the non-sarcopenia group (2-year OS 89.7% versus 59.1%, *P* = 0.03; 2-year RFS 74.9% versus 50.0%, *P* = 0.02). Multivariate analysis revealed that sarcopenia was an independent poor prognostic factor in localized-stage PDAC. Additionally, tumor-infiltrating CD8^+^ T cells in the sarcopenia group were significantly less than in the non-sarcopenia group (*P* = 0.02). However, no difference was observed in driver gene alteration and fib.rotic status. These findings were not observed in advanced-stage PDAC (stage ≥ IIb).

**Conclusions:**

Sarcopenia was associated with a worse prognosis and decreased tumor-infiltrating CD8^+^ T cells in localized-stage PDAC. Sarcopenia may worsen a patient’s prognosis by suppressing local tumor immunity.

**Supplementary Information:**

The online version contains supplementary material available at 10.1245/s10434-023-13569-2.

Pancreatic ductal adenocarcinoma (PDAC) is one of the deadliest cancers, with a 5-year survival of less than 10%.^1^ Cachexia caused by PDAC is associated with a poor prognosis. Cachexia is a complex metabolic disorder characterized by anorexia, unintended weight loss, and wasting of skeletal muscle mass, leading to poor quality of life and mortality.^2^ Additionally, approximately 80% of patients with PDAC develop cachexia by the time they die, and a close relationship between PDAC and cachexia has long been recognized.^2^ Recently, the significance of sarcopenia, a manifestation of cachexia, has been highlighted, and the understanding of its pathogenesis has rapidly advanced.

Sarcopenia is defined as a condition characterized by a progressive decline in skeletal muscle mass and quality^3^ and is found in 30–65% of patients with PDAC at the time of diagnosis.^4^ Several previous studies demonstrated that sarcopenia was significantly associated with a worse long-term prognosis in patients with PDAC and concluded that sarcopenia was an independent poor prognostic factor.^5–7^ However, regarding the impact of sarcopenia on short-term outcomes, including postoperative morbidity, pancreatic fistula, and 30-day mortality, some studies have indicated a correlation,^8,9^ while others have indicated no correlation.^5,10,11^ Thus, the impact of sarcopenia on short-term outcomes is controversial. In addition, no research has clarified the reason for an association between sarcopenia and patient prognosis. Since sarcopenia was reportedly associated with a worse recurrence-free survival (RFS),^6^ the patient’s frailty, a major problem of sarcopenia, including weakness, decreased energy, and lower activity, alone may not explain the poor prognosis in patients with PDAC presenting with sarcopenia. The mechanisms by which sarcopenia leads to a poor prognosis in patients with PDAC are yet to be determined.

Recently, skeletal muscle has attracted attention as an endocrine organ that produces and releases multiple cytokines, known as myokines.^12^ Several studies have suggested that myokines produced by skeletal muscle play a pivotal role in the local immune system in cancers, i.e., tumor-infiltrating lymphocytes (TILs) and tumor-associated macrophages.^13,14^ Sarcopenia may be involved in forming the immunosuppressive tumor microenvironment (TME) in PDAC, contributing to a poor prognosis. However, no research has identified the tumor characteristics, including driver gene alterations and TME, in patients with PDAC with sarcopenia.

Therefore, this study aimed to elucidate the tumor characteristics, including the TME, of PDAC-causing sarcopenia by analyzing 162 patients with PDAC who underwent pancreatic surgery.

## Patients and Methods

### Study Design

We retrospectively analyzed 162 patients with PDAC who underwent pancreatic surgery between April 2008 and March 2017 at Kobe University Hospital. We excluded patients with anaplastic carcinoma, adenosquamous carcinoma, adenocarcinoma derived from intraductal papillary mucinous neoplasms or mucinous carcinoma, and those without available surgical specimens from this study.

We collected clinical information from the medical chart. Patient background information included age, sex, body mass index (BMI), body weight loss of greater than 10% within 6 months, performance status, serum levels of total protein and albumin, neutrophil–lymphocyte ratio (NLR), carcinoembryonic antigen (CEA), carbohydrate antigen 19-9 (CA19-9), family history, alcohol consumption, smoking history, diabetes mellitus (DM), concomitant intraductal papillary mucinous neoplasm (IPMN), and chronic pancreatitis. The laboratory data analyzed in this study were from within 3 months before pancreatic surgery. NLR, an indicator of systemic immune status, was calculated by dividing neutrophil count (/μl) by lymphocyte count (/μl). In addition, the tumor characteristics included tumor size, pathological stage according to the 8th edition of the Union for International Cancer Control,^15^ tumor locations, histological grade, residual tumor status, neoadjuvant chemotherapy, adjuvant chemotherapy, and preoperative radiation. The operation-related information included surgical approach, operation time, estimated blood loss, postoperative morbidity according to the Clavien–Dindo classification (CD),^16^ postoperative pancreatic fistula (POPF) on the basis of the International Study Group of Pancreatic Fistula (ISGPF),^17^ postoperative hospital stay, mortality within 30 days, and reoperation within 30 days. All patients who underwent pancreatic surgery received the same perioperative management as described previously.^18^

The Kobe University Clinical Research Ethical Committee approved this study (approval no. 180235). The ethical committee waived informed consent because of its retrospective nature. The study information was disclosed online, enabling enrolled patients to opt out.

### Definition of Sarcopenia

**Fig. 1:**
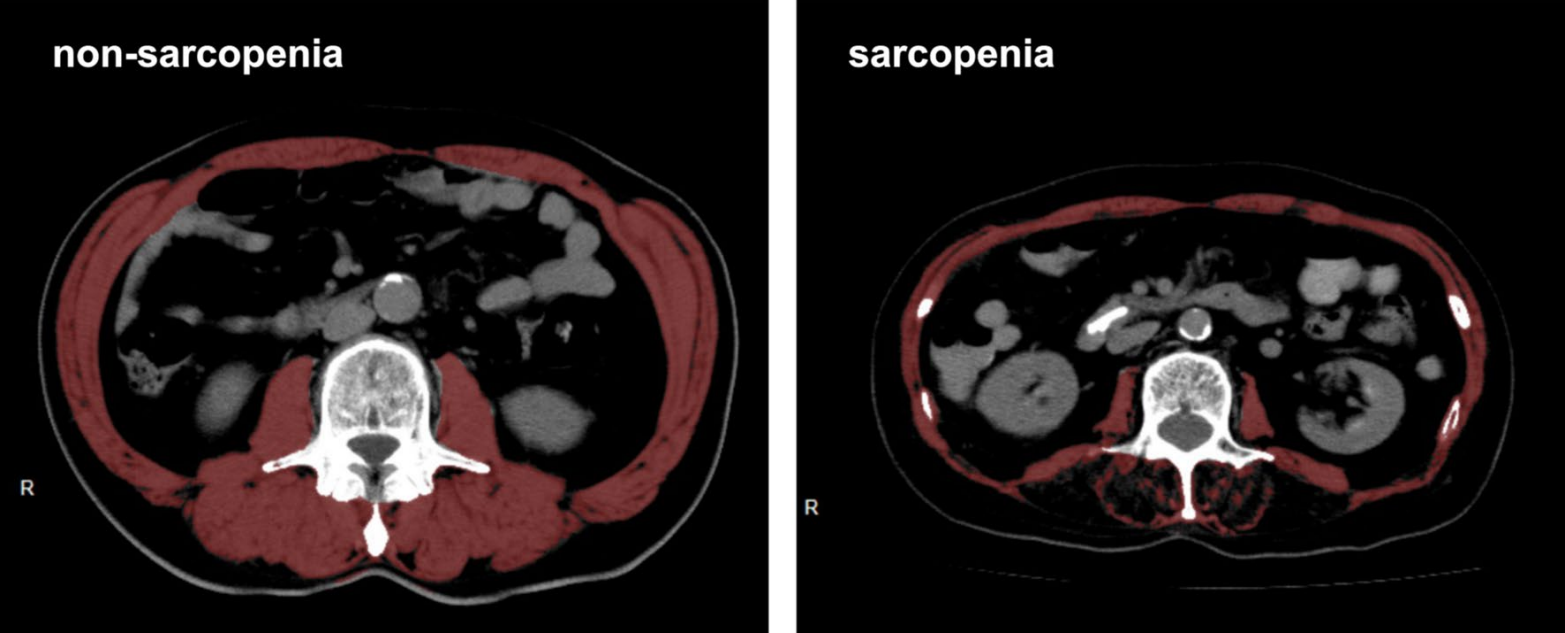
The cross-sectional computed tomographic images at the third lumbar vertebral body level in non-sarcopenic and sarcopenic patients; the red area indicates the total skeletal muscle area; skeletal muscle index (SMI) is the cross-sectional area of the total skeletal muscle/the square of the patient’s height (cm^2^/m^2^)

Sarcopenia was assessed using skeletal muscle index (SMI, cm^2^/m^2^) at the level of the third lumbar vertebral body (L3).^19^ The cross-sectional area of the total skeletal muscle (cm^2^) at the L3 level was measured by analyzing axial CT images using a Ziostation 2 type1000 (Ziosoft, Tokyo, Japan) (Fig. 1). Furthermore, SMI was calculated by dividing the measured area (cm^2^) by the body height squared (m^2^). All CT images analyzed in this study were taken within 3 months before pancreatic surgery.

Sarcopenia was defined as an SMI lower than the sex-specific median value. Therefore, male (*n* = 45) and female (*n* = 36) patients with an SMI lower than the median value of 41.9 cm^2^/m^2^ and 36.6 cm^2^/m^2^, respectively, were assigned to the sarcopenia group (*n* = 81).^20^ The remaining patients were assigned to the non-sarcopenia group (*n* = 81).

## *Immunohistochemistry *(*IHC*)

We used five-micron-thick tissue sections from formalin-fixed paraffin-embedded samples to perform IHC and Elastica van Gieson (EVG) staining using the tissue sections and a VENTANA BenchMark GX (Roche Diagnostics, Basel, Switzerland). The primary antibodies used for IHC and EVG staining included p53 (sc-47698, Santa Cruz Biotechnology, Dallas, TX, USA), CDKN2A/p16 (6695221001, Roche Diagnostics), SMAD4 (sc-7966, Santa Cruz Biotechnology), CD4 (CD4-368-L-CE, Leica Biosystems, Wetzlar, Germany), CD8 (5493846001, Roche Diagnostics), FOXP3 (ab20034, Abcam, Cambridge, UK), and Elastic Stain Kit (ab150667, Abcam).

### Evaluation of Four Driver Gene Alterations

We extracted DNA from the same tissue sections as described previously to perform next-generation sequencing (NGS) analyses and copy number variation (CNV) detection in the same method.^21^ This study determined *KRAS*, *TP53*, *CDKN2A*/p16, and *SMAD4* gene alterations using NGS, CNV detection, and IHC, as reported previously.^22^ Briefly, *KRAS* mutation was determined on the basis of NGS alone. *TP53* alteration was determined using a combination of NGS, CNV detection, and IHC. Furthermore, *CDKN2A*/p16 and *SMAD4* alterations were determined using IHC. Two experienced pathologists (M.K. and T.I.), who were both unaware of the clinical data, reviewed all slides to evaluate the IHC sections. *TP53*, *CDKN2A*/p16, and *SMAD4* assessments demonstrated high concordance between the two pathologists with Kappa values of 0.982 (*P* < 0.0001), 0.964 (*P* < 0.0001), and 0.942 (*P* < 0.0001), respectively.

### Evaluation of TME

This study assessed tumor-stromal collagen, tertiary lymphoid structures (TLSs), and TILs, including CD4^+^ helper T cells, CD8^+^ cytotoxic T cells, and FOXP3^+^ regulatory T cells, as TMEs of PDAC.

Each type of TIL was counted using IHC and ImageJ software (a Java image processing program inspired by the National Institutes of Health, USA).^23^ All 162 cases were categorized into high- and low-density groups on the basis of the median value.

TLSs are organized aggregates of immune cells in nonlymphoid tissues and reportedly contribute to intratumoral immune response.^24^ TLSs were evaluated using hematoxylin and eosin staining sections. One or more TLSs formed within and around the tumor were defined as present TLSs.

The tumor-stromal collagen was assessed using EVG-staining, a technique specifically designed for collagen staining. EVG-stained sections were digitally scanned and analyzed using Adobe Photoshop CC2019 software (Adobe Inc., San Jose, CA). Additionally, tumor-stromal collagen and whole stromal areas were extracted and quantified as the number of pixels. The tumor-stromal collagen proportion was calculated by dividing the tumor-stromal collagen area by the whole stromal area. All 162 cases were classified into high- and low-collagen groups on the basis of the median value.

### Statistical Analysis

All statistical analyses were performed using IBM SPSS Statistics for Windows, version 28.0 (IBM Corp., Armonk, NY, USA). The categorical and continuous data were statistically compared using the chi-squared test (or Fisher’s exact test) and a two-tailed *t*-test, respectively. Overall survival (OS) and RFS were estimated using the Kaplan–Meier method and compared using a log-rank test. Hazard ratios (HRs) and the corresponding 95% confidence intervals (CIs) were estimated using Cox proportional hazard models. Two multivariate models were employed: the first of which was adjusted for residual tumor status, adjuvant chemotherapy, and sarcopenia, and the second which was adjusted for postoperative morbidity, POPF, and sarcopenia. Statistical significance was set at *P* < 0.05.

## Results

### Patient Characteristics with Sarcopenia and Non-Sarcopenia

Patient background information between the sarcopenia and non-sarcopenia groups is presented in Table 1.

**Table 1:** Differences in the patient background between sarcopenic and non-sarcopenic patients

	All cases *N* = 162		Non-sarcopenia *N* = 81		Sarcopenia *N* = 81		*P* value
Age, median (range)							0.08
	69 (40–85)		67 (40–84)		70 (48–85)		
Sex							0.99
Female	72	(44.4%)	36	(44.4%)	36	(44.4%)	
Male	90	(55.6%)	45	(55.6%)	45	(55.6%)	
BMI (kg/m^2^), median ± s.d.							< 0.001
	21.4 ± 3.25		21.9 ± 3.47		20.1 ± 2.59		
Body weight loss							0.49
Absent	83	(51.2%)	44	(54.3%)	39	(48.1%)	
Present	43	(26.5%)	20	(24.7%)	23	(28.4%)	
Unknown	36	(22.2%)	17	(21.0%)	19	(23.5%)	
Performance status							0.69
0	130	(80.2%)	65	(80.2%)	65	(80.2%)	
1	28	(17.3%)	15	(18.5%)	13	(16.0%)	
2	3	(1.9%)	1	(1.2%)	2	(2.5%)	
3	1	(0.6%)	0	(0.0%)	1	(1.2%)	
Total protein (g/dl), mean ± s.d.							0.86
	6.99 ± 0.66		6.99 ± 0.71		6.98 ± 0.60		
Albumin (g/dl), mean ± s.d.							0.96
	3.81 ± 0.49		3.81 ± 0.46		3.81 ± 0.51		
CEA (ng/ml), mean ± s.d.							0.69
	5.33 ± 8.73		5.60 ± 9.94		5.06 ± 7.37		
CA19-9 (U/ml), mean ± s.d.							0.39
	721.0 ± 1837.9		844.7 ± 1993.8		597.3 ± 1670.1		
Family history							0.99
Absent	148	(91.4%)	74	(91.4%)	74	(91.4%)	
Present	14	(8.6%)	7	(8.6%)	7	(8.6%)	
Alcohol consumption							0.19
< 50 g/day	146	(90.1%)	70	(86.4%)	76	(93.8%)	
≥ 50 g/day	16	(9.9%)	11	(13.6%)	5	(6.2%)	
Smoking history							< 0.001
Absent	84	(51.9%)	30	(37.0%)	54	(66.7%)	
Present	78	(48.1%)	51	(63.0%)	27	(33.3%)	
Diabetes mellitus							0.75
Absent	98	(60.5%)	50	(61.7%)	48	(59.3%)	
Present	64	(39.5%)	31	(38.3%)	33	(40.7%)	
IPMN							0.86
Absent	137	(86.7%)	68	(87.2%)	69	(86.3%)	
Present	21	(13.3%)	10	(12.8%)	11	(13.7%)	
Chronic pancreatitis							N/A
Absent	162	(100.0%)	81	(100.0%)	81	(100.0%)	
Present	0	(0.0%)	0	(0.0%)	0	(0.0%)	
Tumor size (mm), mean ± s.d.							0.55
	29.6 ± 10.4		29.1 ± 9.17		30.1 ± 11.6		
Pathological stage^#^							0.86
Localized (≤ IIa)	43	(26.5%)	21	(25.9%)	22	(27.2%)	
Advanced (≥ IIb)	119	(73.5%)	60	(74.1%)	59	(72.8%)	
Tumor location							0.22
Head	117	(72.2%)	62	(76.5%)	55	(67.9%)	
Body/tail	45	(27.8%)	19	(23.5%)	26	(32.1%)	
Histological grade							0.22
Well/moderately differentiated	143	(88.3%)	74	(91.4%)	69	(85.2%)	
Poorly differentiated	19	(11.7%)	7	(8.6%)	12	(14.8%)	
Residual tumor status							0.29
R0	118	(72.8%)	62	(76.5%)	56	(69.1%)	
R1	44	(27.2%)	19	(23.5%)	25	(30.9%)	
R2	0	(0.0%)	0	(0.0%)	0	(0.0%)	
Neoadjuvant chemotherapy							0.62
Absent	144	(88.9%)	71	(87.7%)	73	(90.1%)	
Present	18	(11.1%)	10	(12.3%)	8	(9.9%)	
Adjuvant chemotherapy							0.86
Absent	45	(27.8%)	22	(27.2%)	23	(28.4%)	
Present	117	(72.2%)	59	(72.8%)	58	(71.6%)	
Completed adjuvant chemotherapy							0.44
Yes	24	(51.2%)	11	(54.3%)	13	(48.1%)	
No	86	(26.5%)	47	(24.7%)	39	(28.4%)	
Unknown	7	(22.2%)	1	(21.0%)	6	(23.5%)	
Preoperative radiation							
Absent	161	(99.4%)	81	(100.0%)	80	(98.8%)	0.99
Present	1	(0.6%)	0	(0.0%)	1	(1.2%)	
SMI (cm^2^/m^2^), mean ± s.d.							
Male	42.2 ± 6.75		47.0 ± 5.49		37.3 ± 3.66		< 0.001
Female	37.1 ± 7.08		42.9 ± 4.68		31.3 ± 3.25		< 0.001

*BMI* body mass index, *CEA* carcinoembryonic antigen, *CA19-9* carbohydrate antigen 19-9, *IPMN* intraductal papillary mucinous neoplasm, *SMI* skeletal muscle mass index, *s.d.* standard deviation, *N/A* not applicable

^#^Pathological stage was classified according to the Union for International Cancer Control 8th edition.

BMI, the proportion of patients with a smoking history, and SMI were significantly lower in the sarcopenia group than in the non-sarcopenia group (BMI, *P* < 0.001; smoking history, *P* < 0.001; SMI, *P* < 0.001). There were no significant differences in performance status, body weight loss greater than 10% within 6 months, and nutrition status, including the serum levels of albumin and total protein, between the two groups. No difference was observed in the tumor status, including size, pathological stage, and histological grade, between the two groups.

### Sarcopenia Was an Independent Poor Prognostic Factor in Patients with Localized-Stage PDAC

**Fig. 2:**
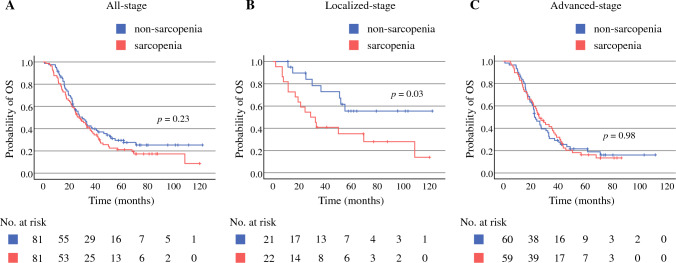
Kaplan–Meier curve of overall survival (OS) after pancreatic surgery according to sarcopenia and non-sarcopenia in patients with all-stage **A**, localized-stage **B**, and advanced-stage **C** pancreatic ductal adenocarcinoma

**Fig. 3:**
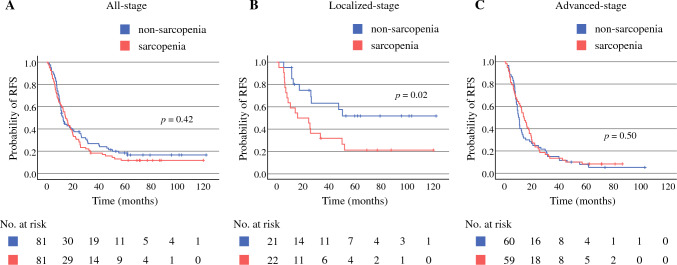
Kaplan–Meier curve of recurrence-free survival (RFS) after pancreatic surgery in sarcopenic and non-sarcopenic patients with all-stage **A**, localized-stage **B**, and advanced-stage **C** pancreatic ductal adenocarcinoma

Figures 2 and 3 show the Kaplan–Meier curves of OS and RFS in the sarcopenic and non-sarcopenic groups, respectively.

The median duration of follow-up was 26.8 (1.1–122.1) months among all patients. Overall, the non-sarcopenia group tended to have a slightly better OS and RFS than the sarcopenia group, although the trends were not statistically significant (2-year OS: 58.3% versus 58.0%, *P* = 0.23; 2-year RFS: 37.7% versus 30.9%, *P* = 0.42) (Figs. 2A, 3A).

We defined pathological stage IIa or lower without metastases, including lymph node metastasis, as a localized stage and other stages as an advanced stage, and subsequently compared the prognoses in each stage to clarify the different effects of sarcopenia on PDAC progression. In localized-stage PDAC, the non-sarcopenia group had a significantly favorable OS and RFS compared with the sarcopenia group (2-year OS: 89.7% versus 59.1%, *P* = 0.03; 2-year RFS: 74.9% versus 50.0%, *P* = 0.02) (Figs. 2B and 3B). However, in advanced-stage PDAC, no difference was found in OS and RFS between the non-sarcopenia and sarcopenia groups (2-year OS: 48.2% versus 57.6%, *P* = 0.98; 2-year RFS: 25.0% versus 23.7%, *P* = 0.50) (Figs. 2C, 3C).

Furthermore, we conducted univariate and multivariate analyses to investigate the relative contributions of each variable to the OS in localized-stage PDAC. The results are presented in Table 2. In the univariate analysis, a residual tumor status of R1 or R2 (HR 4.15, 95% CI 1.65–10.47, *P* < 0.001) and sarcopenia (HR 2.50, 95% CI 1.06–5.89, *P* = 0.04) were extracted as poor prognostic factors after pancreatic surgery. Moreover, we performed multivariate analysis for the three variables, well-known prognostic factors with *P* < 0.200 and sarcopenia,^25–27^ identifying a residual tumor status of R1 or R2 (HR 5.22, 95% CI 1.98–13.80, *P* < 0.001) and sarcopenia (HR 2.58, 95% CI 1.06–6.25, *P* = 0.04) as independent poor prognostic factors after pancreatic surgery.

**Table 2:** Univariate and multivariate analyses of overall survival time in localized-stage PDAC (Cox proportional hazard model)

Variables	No. of patients (%)	Univariate analyses			Multivariate analyses		
		Hazard ratio (95% CI)		*P* value	Hazard ratio^#^ (95% CI)		*P* value
*Age (years)*							
< 65	10 (23.3%)						
≥ 65	33 (76.7%)	1.54 (0.57–4.15)		0.37			
*BMI (kg/m*^*2*^*)*							
< 25	40 (93.0%)						
≥ 25	3 (7.0%)	0.87 (0.20–3.75)		0.85			
*Body weight loss*							
Absent	25 (78.1%)						
Present	7 (21.9%)	1.95 (0.67–5.64)		0.22			
*Performance status*							
0	34 (79.1%)						
≥ 1	9 (20.9%)	1.45 (0.57–3.69)		0.44			
*Sex*							
Female	21 (48.8%)						
Male	22 (51.2%)	0.97 (0.43–2.17)		0.94			
*CEA*							
≤ 5.0	31 (72.1%)						
> 5.0	12 (27.9%)	0.65 (0.24–1.74)		0.68			
*CA19-9*							
≤ 37	16 (37.2%)						
> 37	27 (62.8%)	1.29 (0.55–3.03)		0.68			
*Family history*							
Absent	39 (90.7%)						
Present	4 (9.3%)	0.97 (0.048–2.72)		0.36			
*Alcohol consumption*							
< 50 g/day	40 (93.0%)						
≥ 50 g/day	3 (7.0%)	1.27 (0.37–4.34)		0.70			
*Smoking history*							
Absent	23 (53.5%)						
Present	20 (46.5%)	0.96 (0.43–2.16)		0.93			
*Diabetes mellitus*							
Absent	24 (55.8%)						
Present	19 (44.2%)	0.84 (0.37–1.90)		0.68			
*IPMN*							
Absent	33 (76.7%)						
Present	10 (23.3%)	1.31 (0.48–3.56)		0.68			
*Tumor size*							
< 20	17 (60.5%)						
≥ 20	26 (39.5%)	1.85 (0.76–4.50)		0.18			
*Tumor location*							
Body/tail	12 (27.9%)						
Head	31 (72.1%)	2.35 (0.80–6.89)		0.09			
*Histological grade*							
Well/moderately differentiated	42 (97.7%)						
Poorly differentiated	1 (2.3%)	0.03 (0.00–16.68)		0.28			
*Residual tumor status*							
R0	35 (81.4%)						
R1 or R2	8 (18.6%)	4.15 (1.65–10.47)		< 0.001	5.22	(1.97–13.8)	< 0.001
*Neoadjuvant chemotherapy*							
Absent	42 (97.7%)						
Present	1 (2.3%)	0.05 (0.00–1015.4)		0.55			
*Adjuvant chemotherapy*							
Absent	11 (25.6%)						
Present	32 (74.4%)	0.48 (0.19–1.21)		0.12	0.47	(0.18–1.21)	0.12
*Sarcopenia*							
Absent	22 (51.2%)						
Present	21 (48.8%)	2.50 (1.06–5.89)		0.04	2.58	(1.06–6.25)	0.04

*PDAC* pancreatic ductal adenocarcinoma, *BMI* body mass index, *CEA* carcinoembryonic antigen, *CA19-9* carbohydrate antigen 19-9, *IPMN* intraductal papillary mucinous neoplasm, *SMI* skeletal muscle mass index ^#^The hazard ratio was adjusted for residual tumor status, adjuvant chemotherapy, and sarcopenia.

### Sarcopenia Did Not Worsen Operative and Postoperative Outcomes

The operative and postoperative outcomes between the sarcopenia and non-sarcopenia groups are presented in Table 3 and Supplemental Table 1.

**Table 3:** Differences in operative and postoperative outcomes between sarcopenic and non-sarcopenic patients

	All cases *N* = 162	Non-sarcopenia *N* = 81	Sarcopenia *N* = 81	*P* value
Operative procedure				0.33
Pancreaticoduodenectomy	117 (72.2%)	62 (76.5%)	55 (67.9%)	
Distal pancreatectomy	44 (27.2%)	19 (23.5%)	25 (30.9%)	
Total pancreatectomy	1 (0.6%)	0 (0.0%)	1 (1.2%)	
Surgical approach				0.99
Open	160 (98.8%)	80 (98.8%)	80 (98.8%)	
Laparoscopy	2 (1.2%)	1 (1.2%)	1 (1.2%)	
Operation time (min), median (range)				0.40
	544 (208–833)	545 (208–741)	527 (244–833)	
Estimated blood loss (ml), median (range)				0.91
	655 (0–2834)	650 (0–2834)	660 (80–2560)	
Postoperative morbidity (CD ≥ III)				0.25
Absent	128 (79.0%)	61 (75.3%)	67 (82.7%)	
Present	34 (21.0%)	20 (24.7%)	14 (17.3%)	
POPF^#^ (grade B or C)				0.29
Absent	135 (83.3%)	65 (80.2%)	70 (86.4%)	
Present	27 (16.7%)	16 (19.8%)	11 (13.6%)	
Postoperative hospital stay (days), median (range)				0.03
	23 (7–151)	24 (11–151)	21 (7–75)	
Mortality within 30 days				N/A
Absent	162 (100.0%)	81 (100.0%)	81 (100.0%)	
Present	0 (0.0%)	0 (0.0%)	0 (0.0%)	
Reoperation within 30 days				0.25
Absent	159 (98.1%)	78 (96.3%)	81 (100.0%)	
Present	3 (1.9%)	3 (3.7%)	0 (0.0%)	

*CD* Clavien–Dindo classification, *POPF* postoperative pancreatic fistula, *ISGPF* International Study Group of Pancreatic Fistula, *N/A* not applicable ^#^POPF was diagnosed according to the ISGPF definition

For the operative outcomes, the operative procedure, surgical approach, operation time, and estimated blood loss did not significantly differ between the two groups.

For the postoperative outcomes, the incidence of postoperative morbidity of CD grade 3 or higher and POPF grade B or C was similar between the two groups. However, the duration of postoperative hospital stay was slightly shorter in the sarcopenia group than in the non-sarcopenia group (non-sarcopenia median 24 days versus sarcopenia median 21 days, *P* = 0.03).

Furthermore, we conducted multivariate analyses to investigate whether postoperative outcomes were confounders between sarcopenia and OS in localized-stage PDAC. The results are presented in Supplementary Table 2. These analyses revealed that sarcopenia was a prognostic predictor independent of postoperative outcome variables (HR 3.08, 95% CI 1.24–7.61, *P* = 0.02).

### ***Sarcopenia Was Associated with Decreased Tumor-Infiltrating CD8***^***+***^*** T cells in Localized-Stage PDAC***

Our results indicated no apparent patient frailty that led to a poor prognosis. However, we further investigated the tumor characteristics of PDAC, including TME, to clarify why sarcopenia worsens patient prognosis in localized-stage PDAC. Tumor characteristics of PDAC, including four driver gene alterations, immune status, and tumor-stromal collagen, between the sarcopenia and non-sarcopenia groups are presented in Table 4.

**Table 4:** Different tumor characteristics of PDAC, including gene alteration and immune status, between sarcopenic and non-sarcopenic patients

	All cases		Localized-stage PDAC				*P* value	Advanced-stage PDAC				*P* value
			Non-sarcopenia		Sarcopenia			Non-sarcopenia		Sarcopenia		
	*N* = 162		*N* = 21		N = 22			*N* = 60		*N* = 59		
*KRAS mutatio*							0.66					0.99
Absent	12	(7.4%)	3	(14.3%)	2	(9.1%)		4	(6.7%)	3	(5.1%)	
Present	150	(92.6%)	18	(85.7%)	20	(90.9%)		56	(93.3%)	56	(94.9%)	
G12D	56	(37.3%)	8	(44.4%)	2	(10.0%)		19	(31.7%)	27	(45.8%)	
G12V	51	(34.0%)	3	(16.7%)	8	(40.0%)		21	(35.0%)	19	(32.2%)	
G12R	28	(18.7%)	5	(27.8%)	6	(30.0%)		11	(18.3%)	6	(10.2%)	
Q61H	9	(6.0%)	1	(5.6%)	3	(15.0%)		3	(5.0%)	2	(3.4%)	
Others	6	(4.0%)	1	(5.6%)	1	(5.0%)		2	(3.3%)	2	(3.4%)	
*TP53* alteration							0.82					0.06
Absent	51	(31.5%)	6	(28.6%)	7	(31.8%)		24	(40.0%)	14	(23.7%)	
Present	111	(68.5%)	15	(71.4%)	15	(68.2%)		36	(60.0%)	45	(76.3%)	
*CDKN2A*/p16 alteration							0.44					0.74
Absent	58	(35.8%)	12	(57.1%)	10	(45.5%)		19	(31.7%)	17	(28.8%)	
Present	104	(64.2%)	9	(42.9%)	12	(54.5%)		41	(68.3%)	42	(71.2%)	
*SMAD4* alteration							0.99					0.40
Absent	100	(61.7%)	16	(76.2%)	16	(72.7%)		32	(53.3%)	36	(61.0%)	
Present	62	(38.3%)	5	(23.8%)	6	(27.3%)		28	(46.7%)	23	(39.0%)	
CD4^+^ TILs							0.16					0.78
Low	82	(50.6%)	7	(33.3%)	12	(54.5%)		31	(51.7%)	32	(54.2%)	
High	80	(49.4%)	14	(66.7%)	10	(45.5%)		29	(48.3%)	27	(45.8%)	
CD8^+^ TILs							0.02					0.52
Low	81	(50.0%)	7	(33.3%)	15	(68.2%)		28	(46.7%)	31	(52.5%)	
High	81	(50.0%)	14	(66.7%)	7	(31.8%)		32	(53.3%)	28	(47.5%)	
FOXP3^+^ TILs							0.65					0.65
Low	81	(50.0%)	10	(47.6%)	12	(54.5%)		31	(51.7%)	28	(47.5%)	
High	81	(50.0%)	11	(52.4%)	10	(45.5%)		29	(48.3%)	31	(52.5%)	
TLSs							0.99					0.93
Absent	50	(30.9%)	5	(23.8%)	5	(22.7%)		19	(31.7%)	21	(35.6%)	
Present	112	(69.1%)	16	(76.2%)	17	(77.3%)		41	(68.3%)	38	(64.4%)	
NLR, mean ± s.d							0.54					0.38
	2.26 ± 0.81		2.30 ± 0.58		2.18 ± 0.67			2.20 ± 0.80		2.34 ± 0.94		
Tumor-stromal collagen							0.44					0.65
Low	81	(50.0%)	12	(57.1%)	10	(45.5%)		31	(51.7%)	28	(47.5%)	
High	81	(50.0%)	9	(42.9%)	12	(54.5%)		29	(48.3%)	31	(52.5%)	

*PDAC*, pancreatic ductal adenocarcinoma; *TILs*, tertiary lymphoid structures; *NLR*, neutrophil-lymphocyte ratio; *TLS*, tertiary lymphoid structures

No difference was observed in *KRAS* mutation, including mutated allele frequencies, *TP53* alteration, *CDKN2A*/p16 alteration, and *SMAD4* alteration, despite the stage of PDAC.

For local immune status, in localized-stage PDAC, CD8^+^ TILs were significantly lower in the sarcopenia group than in the non-sarcopenia group (66.7% versus 31.8%, *P* = 0.02). In addition, CD4^+^ TILs also tended to be lower in the sarcopenia group than in the non-sarcopenia group (66.7% versus 45.5%, *P* = 0.16). No significant difference was observed in FOXP3^+^ TILs (*P* = 0.65) and TLSs (*P* = 0.99) between the two groups. However, these differences were not found in advanced-stage PDAC.

No significant difference was observed in NLR and the amount of tumor-stromal collagen between the sarcopenia and non-sarcopenia groups.

## Discussion

This study demonstrated that the sarcopenia group had a significantly worse prognosis than the non-sarcopenia group in localized-stage PDAC, with no lymph node metastasis. A previous meta-analysis, including both palliative and curative surgeries, showed that sarcopenia was associated with an increased hazard of death (HR 1.35, 95% CI 1.18–1.54).^28^ However, some studies have shown that patients with and without sarcopenia had no significant difference in long-term prognosis.^29,30^ The authors argued that different measurements, including SMI, total psoas index, and total psoas volume, could explain this from each study. However, these studies did not examine the prognosis stratified according to the stage of PDAC. Our results might provide an answer to these discrepancies.

We showed that sarcopenia did not negatively affect the operative and postoperative outcomes in patients with PDAC. In addition, sarcopenia was significantly associated with a shorter RFS in localized-stage PDAC. Several previous studies and meta-analyses have demonstrated no significant difference in perioperative outcomes, including morbidity of CD ≥ grade 3, which is consistent with our results.^31^ Some studies have also reported that the RFS was shorter in patients with PDAC with sarcopenia.^6,7^ These findings supported the hypothesis that sarcopenia may be associated with the aggressive progression of PDAC, leading to a poor prognosis independent of the patient’s frailty. However, the mechanism linking sarcopenia to the aggressive progression of PDAC is yet to be addressed.

Notably, we demonstrated that sarcopenia was associated with a decrease in tumor-infiltrating T cells, particularly CD8^+^ T cells, in localized-stage PDAC. In the last two decades, skeletal muscle has been increasingly recognized as an endocrine organ that modulates immune function through various myokines, including interleukin (IL) 6, IL-7, and IL-15, or cell-to-cell interaction, rather than a pure locomotor.^32^ Sarcopenia may interfere with these interactions, suppress tumor immunity, and explain the worse outcomes for patients with sarcopenia. However, except for one study on cholangiocarcinoma,^33^ no reports exist regarding the association between sarcopenia and tumor immune status in cancers, including PDAC, using human samples. Therefore, this study is the first to comprehensively analyze the association between sarcopenia, patient prognosis, and tumor immune system in PDAC. Furthermore, since sarcopenia strongly affects a patient’s prognosis in localized-stage PDAC, with no lymph node metastasis, sarcopenia may be primarily involved in immune mechanisms regulating lymph node metastasis.

This study has some limitations. First, whether sarcopenia is a cause or consequence of immunosuppressive TME is inconclusive. Second, the systemic immune status could not be evaluated. Third, no relevant data were identified to explain the difference in the impact of sarcopenia between localized-stage and advanced-stage PDAC. Fourth, this study determined sarcopenia only by the quantity of the skeletal muscle since skeletal muscle function could not be evaluated because of the study’s retrospective nature. To elucidate whether or not sarcopenia genuinely suppresses tumor immunity for PDAC, we need to clarify the tumor characteristics that cause cachexia and sarcopenia and their effects on the immune system in PDAC using animal models, such as patient-derived tumor xenograft models, in a future study.

In conclusion, sarcopenia was associated with a poor prognosis and decreased tumor-infiltrating CD8^+^ T cells in localized-stage PDAC. Sarcopenia may worsen a patient’s prognosis by suppressing local tumor immunity.

## Supplementary Information

Below is the link to the electronic supplementary material

Supplementary file1 (DOCX 25 kb)
