# Supplementary material for: Association of Sarcopenia with a Poor Prognosis and Decreased Tumor-Infiltrating CD8-Positive T Cells in Pancreatic Ductal Adenocarcinoma: A Retrospective Analysis
Source: Ann Surg Oncol. 2023 May 16;30(9):5776–87. doi: 10.1245/s10434-023-13569-2 (PMC10409680; doi:10.1245/s10434-023-13569-2)
Supplement: Supplementary file 1 — Supplementary file1 (DOCX 25 kb) [file 10434_2023_13569_MOESM1_ESM.docx]

**Supplemental Table 1.**  **Differences in operative and postoperative outcomes between sarcopenic and non-sarcopenic patients in localized-stage and advanced-stage PDAC**

|  |  | **All cases** | | **Localized-stage PDAC** | | | |  | **Advanced-stage PDAC** | | | |  |
| --- | --- | --- | --- | --- | --- | --- | --- | --- | --- | --- | --- | --- | --- |
|  |  |  |  | **non-sarcopenia** | | **sarcopenia** | |  | **non-sarcopenia** | | **sarcopenia** | |  |
|  |  | **N = 162** | | **N = 21** | | **N = 22** | | ***P* value** | **N = 60** | | **N = 59** | | ***P* value** |
| Operative procedure | |  |  |  |  |  |  | 0.49 |  |  |  |  | 0.09 |
|  | Pancreaticoduodenectomy | 117 | (72.2%) | 14 | (66.7%) | 16 | (72.7%) |  | 48 | (80.0%) | 39 | (66.1%) |  |
|  | Distal pancreatectomy | 44 | (27.2%) | 7 | (33.3%) | 5 | (22.7%) |  | 12 | (20.0%) | 20 | (33.9%) |  |
|  | Total pancreatectomy | 1 | (0.6%) | 0 | (0.0%) | 1 | (4.5%) |  | 0 | (0.0%) | 0 | (0.0%) |  |
| Surgical approach | |  |  |  |  |  |  | 0.99 |  |  |  |  | 0.99 |
|  | Open | 160 | (98.8%) | 21 | (100.0%) | 21 | (95.5%) |  | 59 | (98.3%) | 59 | (100.0%) |  |
|  | Laparoscopy | 2 | (1.2%) | 0 | (0.0%) | 1 | (4.5%) |  | 1 | (1.7%) | 0 | (0.0%) |  |
| Operation time (min), median (range) | |  |  |  |  |  |  | 0.66 |  |  |  |  | 0.23 |
|  |  | 544 (208-833) | | 535 (216-682) | | 523.5 (279-714) | |  | 560.5 (208-741) | | 527 (244-833) | |  |
| Estimated blood loss (ml), median (range) | |  |  |  |  |  |  | 0.36 |  |  |  |  | 0.68 |
|  |  | 655 (0-2834) | | 650 (100-1308) | | 670 (90-2455) | |  | 650 (0-2834) | | 660 (80-2560) | |  |
| Postoperative morbidity (CD ≥ III) | |  |  |  |  |  |  | 0.28 |  |  |  |  | 0.53 |
|  | Absent | 128 | (79.0%) | 15 | (71.4%) | 19 | (86.4%) |  | 46 | (76.7%) | 48 | (81.4%) |  |
|  | Present | 34 | (21.0%) | 6 | (28.6%) | 3 | (13.6%) |  | 14 | (23.3%) | 11 | (18.6%) |  |
| POPF^#^ (grade B or C) | |  |  |  |  |  |  | 0.24 |  |  |  |  | 0.50 |
|  | Absent | 135 | (83.3%) | 16 | (76.2%) | 20 | (90.9%) |  | 48 | (80.0%) | 50 | (84.7%) |  |
|  | Present | 27 | (16.7%) | 5 | (23.8%) | 2 | (9.1%) |  | 12 | (20.0%) | 9 | (15.3%) |  |
| Postoperative hospital stay (days), median (range) | |  |  |  |  |  |  | 0.21 |  |  |  |  | 0.07 |
|  |  | 23 (7-151) | | 25 (12-116) | | 24.5 (7-53) | |  | 23.5 (11-151) | | 20 (12-75) | |  |
| Mortality within 30 days | |  |  |  |  |  |  | N/A |  |  |  |  | N/A |
|  | Absent | 162 | (100.0%) | 21 | (100.0%) | 22 | (100.0%) |  | 60 | (100.0%) | 59 | (100.0%) |  |
|  | Present | 0 | (0.0%) | 0 | (0.0%) | 0 | (0.0%) |  | 0 | (0.0%) | 0 | (0.0%) |  |
| Re-operation within 30 days | |  |  |  |  |  |  | 0.49 |  |  |  |  | 0.50 |
|  | Absent | 159 | (98.1%) | 20 | (95.2%) | 22 | (100.0%) |  | 58 | (96.7%) | 59 | (100.0%) |  |
|  | Present | 3 | (1.9%) | 1 | (4.8%) | 0 | (0.0%) |  | 2 | (3.3%) | 0 | (0.0%) |  |

PDAC, pancreatic ductal adenocarcinoma; CD, Clavien-Dindo classification; POPF, postoperative pancreatic fistula; ISGPF, International Study Group of Pancreatic Fistula; N/A, not applicable

#POPF was diagnosed according to the ISGPF definition.

**Supplemental Table 2. The relative contribution of postoperative outcomes and sarcopenia to the OS in localized-stage PDAC (Cox proportional hazard model)**

|  |  |  |  | **Univariate analyses** | | | |  | **Multivariate analyses** | | |
| --- | --- | --- | --- | --- | --- | --- | --- | --- | --- | --- | --- |
| **Variables** | | **No. of patients (%)** | | | **Hazard Ratio (95% CI)** | | ***P* value** |  | **Hazard Ratio^#^ (95% CI)** | | ***P* value** |
| Postoperative morbidity (CD ≥ III) | |  |  |  | |  |  |  |  |  |  |
|  | Absent | 34 | (79.1%) |  | |  |  |  |  |  |  |
|  | Present | 9 | (20.9%) | 0.64 | | (0.22-1.89) | 0.42 |  | 0.42 | (0.12-1.44) | 0.17 |
| POPF^*^ (grade B or C) | |  |  |  | |  |  |  |  |  |  |
|  | Absent | 36 | (83.7%) |  | |  |  |  |  |  |  |
|  | Present | 7 | (16.3%) | 0.60 | | (0.18-2.02) | 0.41 |  |  |  |  |
| Re-operation within 30 days | |  |  |  | |  |  |  |  |  |  |
|  | Absent | 42 | (97.7%) |  | |  |  |  |  |  |  |
|  | Present | 1 | (2.3%) | 5.38 | | (0.66-43.77) | 0.12 |  | 23.25 | (1.89-286.33) | 0.01 |
| Sarcopenia | |  |  |  | |  |  |  |  |  |  |
|  | Absent | 22 | (51.2%) |  | |  |  |  |  |  |  |
|  | Present | 21 | (48.8%) | 2.50 | | (1.06-5.89) | 0.04 |  | 3.08 | (1.24-7.61) | 0.02 |

#The hazard ratio was adjusted for postoperative morbidity, re-operation within 30 days, and sarcopenia.

*POPF was diagnosed according to the ISGPF definition.

OS, overall survival; PDAC, pancreatic ductal adenocarcinoma; CD, Clavien-Dindo classification; POPF, postoperative pancreatic fistula; ISGPF, International Study Group of Pancreatic Fistula
